# Optical clearing potential of immersion-based agents applied to thick mouse brain sections

**DOI:** 10.1371/journal.pone.0216064

**Published:** 2019-05-10

**Authors:** Mathew Loren, Christian Crouzet, Adrian Bahani, Vitaly Vasilevko, Bernard Choi

**Affiliations:** 1 Department of Biomedical Engineering, University of California, Irvine, California, United States of America; 2 Beckman Laser Institute and Medical Clinic, University of California, Irvine, California, United States of America; 3 Institute for Memory Impairments and Neurological Disorders, University of California, Irvine, California, United States of America; 4 Department of Surgery, University of California, Irvine, California, United States of America; 5 Edwards Lifesciences Center for Advanced Cardiovascular Technology, University of California, Irvine, California, United States of America; Texas A&M University, UNITED STATES

## Abstract

We have previously demonstrated that the use of a commercially-available immersion-based optical clearing agent (OCA) enables, within 3–6 hours, three-dimensional visualization of subsurface exogenous fluorescent and absorbing markers of vascular architecture and neurodegenerative disease in thick (0.5–1.0mm) mouse brain sections. Nonetheless, the relative performance of immersion-based OCAs has remained unknown. Here, we show that immersion of brain sections in specific OCAs (FocusClear, RIMS, sRIMS, or Sca*l*eSQ) affects both their transparency and volume; the optical clearing effect occurs over the entire visible spectrum and is reversible; and that Sca*l*eSQ had the highest optical clearing potential and increase in imaging depth of the four evaluated OCAs, albeit with the largest change in sample volume and a concomitant decrease in apparent microvascular density of the sample. These results suggest a rational, quantitative framework for screening and characterization of the impact of optical clearing, to streamline experimental design and enable a cost-benefit assessment.

## Introduction

Imaging thicker tissue sections is limited due to light scattering [1]. Recent progress in optical clearing methods modulates optical scattering to enable volumetric imaging without the need for sectioning. Entire organs can be cleared and imaged with minimal damage to structures of interest and without requiring extensive post-processing of data. Several optical clearing approaches, including variants of CLARITY [2,3], variants of Sca*l*e [4], FocusClear [5,6], RIMS/sRIMS [7], variants of 3DISCO [8], and SeeDB [9], are reported to reduce scattering within biological tissues and enable visualization of subsurface tissue structures, with a major emphasis on the brain.

These approaches differ considerably in terms of sample preparation, time required for clearing, researcher-time required during the clearing process, complexity of the clearing protocol, and overall cost. To maximize use of limited resources, researchers should select the most optimal optical clearing approach to suit their individual needs. Although whole-brain optical clearing enables detailed study of long-range connectivity [10], the cost in researcher-hours and materials can be relatively high. Sectioning of the brain into thick (0.5–1.0mm) tissue sections can help reduce this cost by enabling use of immersion-based optical clearing agents (OCAs) and parallel fluorescence labeling and clearing of the brain sections. In recent studies, we found that the use of a commercially-available immersion-based OCA, FocusClear, enables, within 3–6 hours, three-dimensional visualization of subsurface fluorescently-labeled vasculature [6] and exogenous fluorescent and absorbing markers of fibrillar beta-amyloid and cerebral microhemorrhages, respectively [5] in thick (0.5–1.0mm) mouse brain sections. However, the performance of FocusClear versus other immersion-based OCAs remains unknown.

Here, we demonstrate immersion-based optical clearing affects both transparency and volume of brain samples, with the temporal dynamics varying with OCA. Here, we focus on protocols that involve a single immersion step, as opposed to multi-step immersion-based clearing protocols (i.e., CUBIC, TDE, FRUIT, RTF, etc.) [11–15]. Moreover, the imaging depth achieved with confocal microscopy correlates with the reduction in optical attenuation. Together, the work presents a simple framework for quantitative comparison of OCAs.

## Materials and methods

### Animal model

We performed experiments on adult male mice (n = 5, C3H strain, 7–11 weeks old, 25-30g, Charles River Laboratories, Wilmington, MA). This work was in compliance with a protocol approved by the University of California, Irvine Institutional Animal Care and Use Committee.

### Fluorophore

*Lycopersicon esulentum* tomato lectin is a glycoprotein with a binding affinity to glycoprotein moieties that are found in the vascular endothelium of rodents [16]. In this study, we used lectin-Dylight 650 (Vector Laboratories, Inc., Burlingame, CA) to label the vasculature (see below for procedural details).

### Vessel painting

A 200μL dose of lectin-Dylight650 diluted 1:3 with PBS was administered via retro-orbital injection [17] into the microcirculation of mice anesthetized with an intraperitoneal injection of a cocktail of ketamine (90 mg/kg) and xylazine (10mg/kg). The lectin was allowed to circulate for 30min prior to cardiac perfusion.

### Cardiac perfusion

To obtain brain samples with lectin-labeled vasculature, we adapted a procedure first described by Li et al [16] and used subsequently by our group [5,6,18,19]. Briefly, immediately after euthanasia with an overdose of Sodium Pentobarbitol, we opened the chest cavity and performed a cardiac perfusion of saline (to flush blood out of the vasculature) followed by cardiac perfusion of buffered formalin to initiate the fixation process. We then extracted the brain and immersed it in buffered formalin for 24 hours. We then transferred the brain to a PBS with 0.02% Sodium Azide solution to mitigate photodegradation of the fluorophore.

### Preparation of brain sections

**Fig 1:**
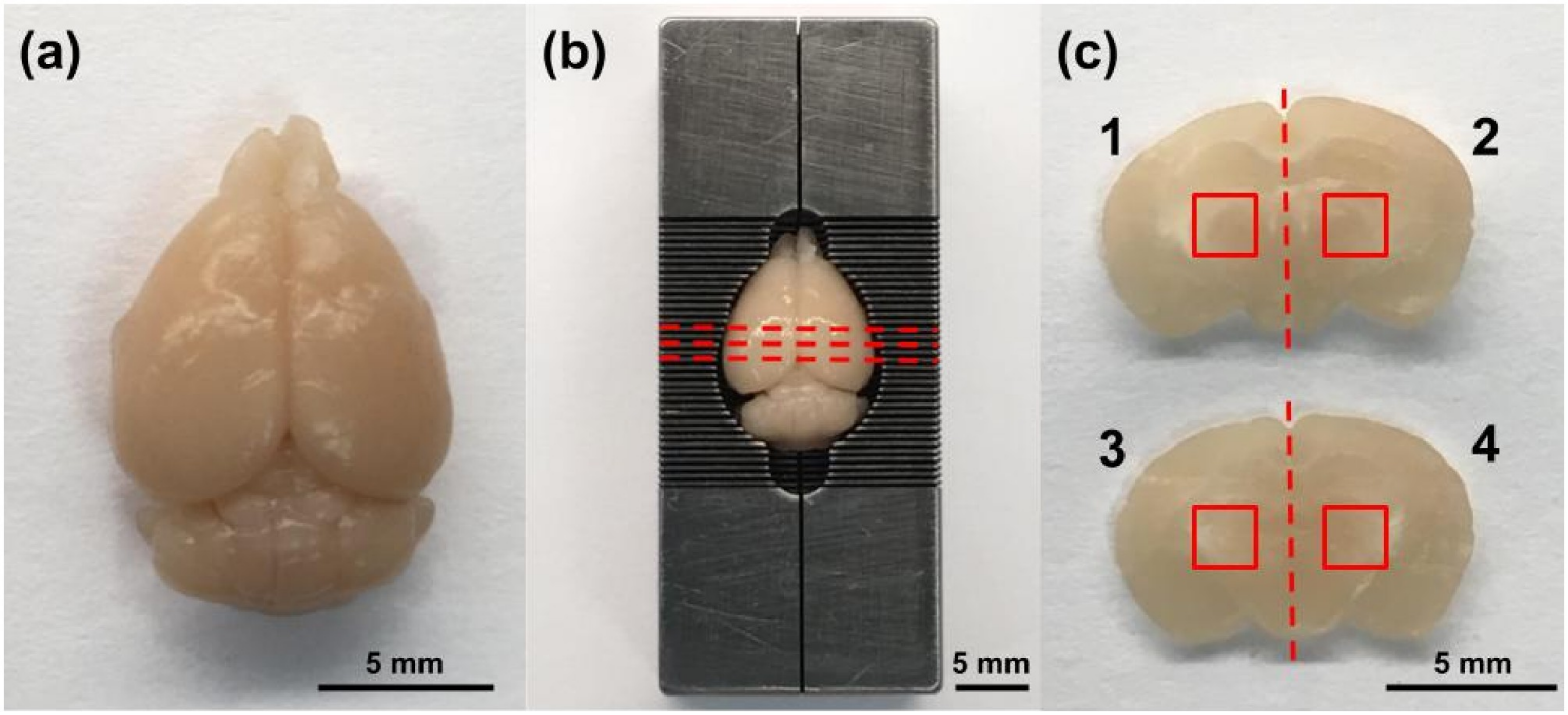
Sample preparation protocol. (a) Brain extracted after cardiac perfusion. (b) Brain placed in mouse brain slicer matrix. (c) Two 1 mm thick coronal sections removed from midpoint of brain. Red dashed lines in (b) denote approximate locations at which each section is removed. Each of the two sections were bisected, resulting in four samples that were then exposed to one of the four optical clearing agents. Red solid lines denote regions of interest probed and analyzed for attenuation measurements and confocal microscopy.

A brain matrix slicer (Zivic Instruments) was used to section each brain into 1-mm-thick coronal samples. Two adjacent coronal sections were taken from the approximate middle of each brain. In total, we studied 10 coronal sections extracted from five brains. To compare the attenuation and physical effects of OCAs, each coronal section was cut into four samples (Fig 1) and randomly assigned to one of the four OCAs tested in this study.

## Optical clearing agents (OCAs)

We studied the following four OCAs: FocusClear (CelExplorer, Taiwan), refractive index matching solution (RIMS), sorbitol RIMS (sRIMS), and Sca*l*eSQ. For RIMS, sRIMS, and Sca*l*eSQ, we followed preparation protocols described in the respective papers with reagents purchased from Sigma Aldrich, Carlsbad, CA:

- *RIMS*: 88% (w/v) Histodenz (PN: S2158-100G) in 0.02 M phosphate buffer solution with 0.01% sodium azide. Bring to 7.5 pH with NaOH [7].
- *sRIMS*: 70% (w/v) sorbitol (PN: 240850) in 0.02 M phosphate buffer solution with 0.01% sodium azide. Bring to 7.5 pH with NaOH [7].
- *Sca*l*eSQ*: 9.1 M urea (PN: U5378), 22.5% (w/v) sorbitol solution, and 4.7% Triton X-100 (PN: X100). Gentle heating required to fully dissolve urea crystals [4].

### Optical attenuation spectroscopy

To estimate the attenuation coefficient of each sample, we adapted a method previously described by d’Esposito et al [20]. We used a custom-built optical device consisting of a microscope light source illuminator (V-Lux-1000, Volpi USA, Auburn, NY), integrating sphere (Labsphere, North Sutton, NH), imaging lens (Optem, Qioptiq Photonics, Munich, Germany), and 12-bit monochrome CCD camera equipped with an electro-optic liquid crystal tunable filter (LCTF) (Nuance, PerkinElmer). The light from the halogen bulb was coupled to the input port (25mm) of the sphere. The light exited the 6mm output port of the sphere positioned at 90 degrees with respect to the input port. The sample holder (described below) was positioned in direct contact with the output port. The transmitted light was spectrally resolved with the LCTF set at a wavelength in the range of 420 to 720nm and imaged with the camera.

### Confocal microscope

For three-dimensional imaging of Dylight650 fluorescence from brain samples, we utilized a commercial confocal microscope (Fluoview FV3000, Olympus, Tokyo, Japan). We used 640nm laser excitation and a 10x objective (Plan-Apo 10x/0.4 WD 3.1mm).

### Clearing and attenuation analysis

Each sample was immersed in a specific OCA for a total of 24 hours. During immersion, the samples were placed in an incubator (37°C). At time points of 0, 3, 6, and 24 hours, optical transmittance data were collected using the optical attenuation spectroscopy device described above. To study reversal of the optical clearing effect, each OCA-treated sample subsequently was immersed in the PBS-sodium azide solution for 24 hours and attenuation data collected. For the attenuation measurements, each sample was removed from the OCA and placed in a custom holder consisting of two glass slides of known thickness. The sample thickness was estimated using a digital micrometer [21]. The holder was placed at the exit port of the integrating sphere and a portion of the transmitted light was collected by the lens attached to the camera. We paired each sample measurement with a maximum transmittance measurement collected using the sample holder containing saline. Using the LCTF, we collected transmittance data at center wavelengths of 420 to 720 nm, with a step size of 10 nm. We used custom-written MATLAB code (R2017b, The MathWorks, Natick, MA) to estimate a spectrum of optical attenuation coefficients [μ(λ)] within a selected region of interest, using Beer’s law:

$$\mu \left(\lambda \right)=-\text{ln}\left[\text{I}\left(\lambda \right)/{\text{I}}_{\text{o}}\left(\lambda \right)\right]/\text{L}$$

where I(λ) and I_o_(λ) are the transmitted spectral intensities measured through the holder containing the brain sample and saline, respectively; and L the measured sample thickness. To calculate normalized attenuation coefficients, we calculated for each sample and each time point, the quotient of μ(λ,t) and μ(λ,t = 0). Similar to our previous work [21], we define the optical clearing potential (OCP) as the quotient of μ(λ,t = 0) and μ(λ,t). In addition, measurements of the lateral dimensions of each sample were collected at each time point with a ruler visible in each frame for calibration of pixel size and binarized images in MATLAB used to estimate surface area.

### Confocal fluorescence microscopic imaging

Samples were imaged using an excitation wavelength of 640 nm and emission wavelength band of 650-750nm. To achieve consistency among samples, imaging was performed within the midbrain/thalamus region of each section, with z-stacks collected starting at the surface of the sample proximal to the objective. For each z-stack, 101 images (512x512 pixel resolution, 2.5μm lateral resolution) were collected with a step size of 10μm, resulting in a total imaging depth of 1mm. Care was taken to control the starting position of the depth scan for each sample, to facilitate comparison of the data collected from each sample. To quantify vascular density, which is a measure of total vessel length per area, we used an algorithm described in our previous publication [22]. To account for the difference in fluorescence emission intensity at each depth, the intensity and size thresholds were changed for each depth but applied equally for images collected with each OCA.

### Statistical analysis

To test various hypotheses (described in the Results section), we used either repeated measures or one-way ANOVA with Holm-Sidak’s multiple comparison tests. All analyses were performed using Prism (Version 7, GraphPad Software, La Jolla, CA).

## Results

### Optical clearing affects both transparency and volume of brain samples

**Fig 2:**
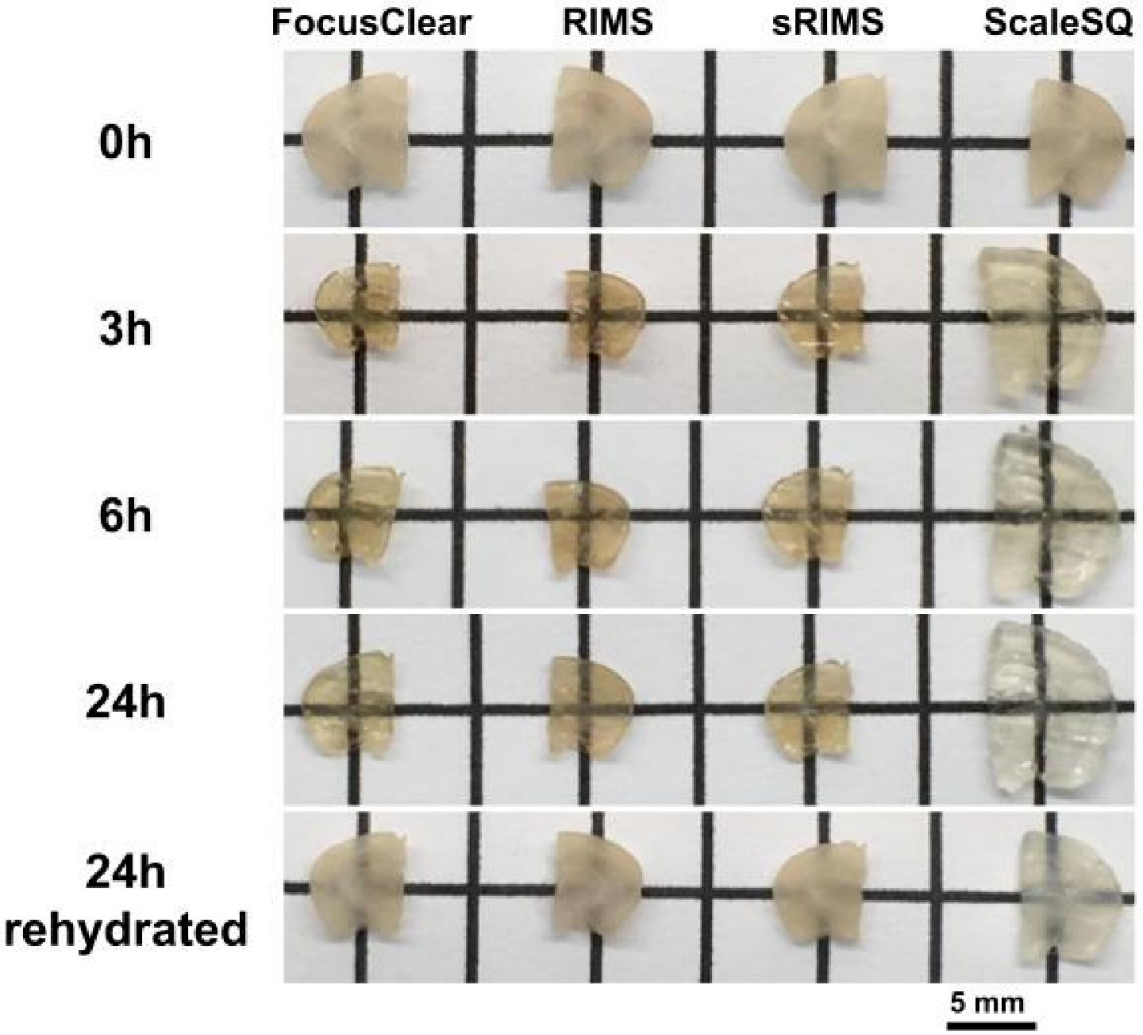
Representative photos of brain samples taken during immersion in each OCA. Time points of 0,3,6, and 24h are shown as well as 24h after re-immersion of samples in PBS-sodium azide.

To investigate the relative effects of immersion-based OCAs on brain samples, we performed qualitative and quantitative inspection of each sample at different time points. Immersion within each OCA increased its transparency (Fig 2). The reversal of clearing effects can be observed after samples were returned to PBS-sodium azide. Samples immersed in FocusClear, RIMS, or sRIMS retained some hue innate to the samples, whereas samples immersed in Sca*l*eSQ became nearly colorless and more translucent than the other samples. Samples immersed in Sca*l*eSQ increased in volume, while samples immersed in each of the other three OCAs decreased in volume during the initial 6h (Table 1). For RIMS and sRIMS, the volume at 24h was greater than the volume at 6h. A comparison of changes in thickness and lateral dimensions suggest that sample expansion and contraction occur in an anisotropic manner, as the data do not follow a quadratic trend (S1 Fig). Immersion in each of the OCAs led to diminished sample integrity, as each sample post immersion required more delicate handling to avoid tearing.

**Table 1:** Change in volume and thickness of brain samples during optical clearing.

	Hour 0	Hour 3	Hour 6	Hour 24	24 Hour Restored
**FocusClear**	100%	Volume: 67% (62, 86) Thickness: 93% (80, 96)	Volume: 78% (62, 88) Thickness: 86% (68, 86)	Volume: 92% (81, 101) Thickness: 86% (69, 92)	Volume: 100% (96, 109) Thickness: 87% (74, 87)
**RIMS**	100%	Volume: 90% (80, 109) Thickness: 94% (93, 97)	Volume: 93% (79, 102) Thickness: 89% (83, 91)	Volume: 110% (97, 117) Thickness: 87% (86, 90)	Volume: 111% (108, 119) Thickness: 91% (88, 93)
**sRIMS**	100%	Volume: 80% (71, 88) Thickness: 98% (96, 101)	Volume: 82% (75, 94) Thickness: 115% (114, 115)	Volume: 97% (90, 102) Thickness: 112% (109, 112)	Volume: 104% (100, 106) Thickness: 107% (107, 112)
**ScaleSQ**	100%	Volume: 120.7% (116.5, 126.1) Thickness: 105% (104, 111)	Volume: 137.7% (134.9, 152.1) Thickness: 116% (111, 126)	Volume: 183.1% (171.7, 199.1) Thickness: 125% (119, 127)	Volume: 110.9% (107.1, 113.8) Thickness: 100% (97, 104)

Volume percentage normalized to initial volume of respective sample before optical clearing. Thickness percentage represents the thickness normalized to initial thickness of respective sample before optical clearing. Values represent medians of data set with minimum and maximum shown in parenthesis.

### Optical clearing achieves a reduction in attenuation of each sample over the visible spectrum

**Fig 3:**
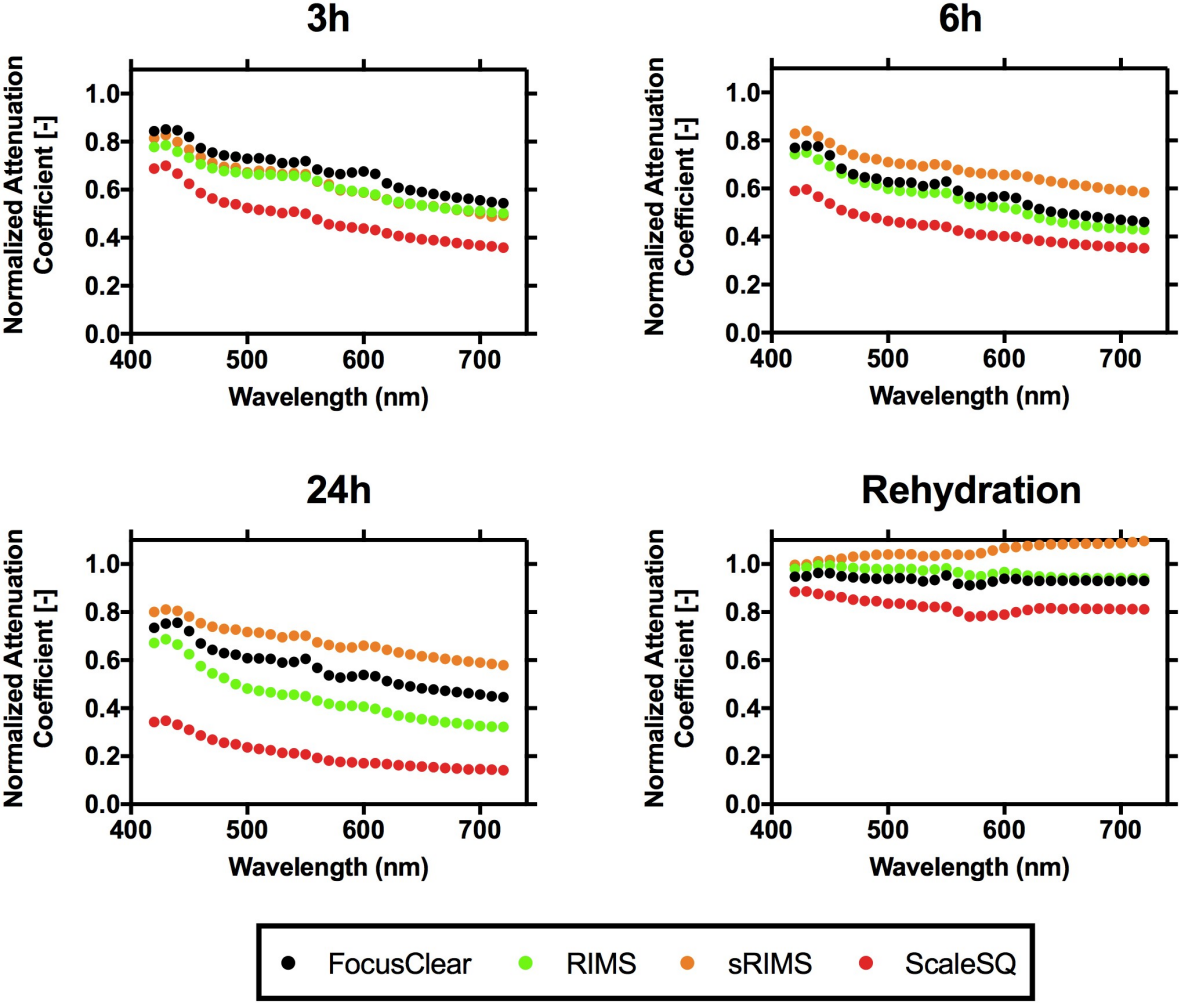
Normalized attenuation coefficients measured for each OCA over the 420–720 nm wavelength range. Data points represent median values of measurements. Error bars representing spread are omitted for clarity.

To investigate quantitative effects of optical clearing, we estimated the attenuation of each sample using transmittance imaging. Due to an error during data collection, one sample of Sca*l*eSQ was omitted from analysis. The median attenuation of light through each sample gradually decreased at each measured wavelength (Fig 3, Table 2).

**Table 2:** Optical attenuation coefficient (λ = 640nm) during optical clearing.

	0 hr	3 hr	6 hr	24 hr	Rehydrated
**FocusClear**	1	0.598 (0.464, 0.622)	0.504 (0.450, 0.624)	0.491 (0.466, 0.506)	0.930 (0.894, 1.087)
**RIMS**	1	0.542 (0.464, 0.578)	0.468 (0.403, 0.601)	0.362 (0.300, 0.379)	0.945 (0.905, 0.981)
**sRIMS**	1	0.542 (0.526, 0.688)	0.630 (0.559, 0.633)	0.624 (0.593, 0.649)	1.082 (1.020, 1.096)
**ScaleSQ**	1	0.441 (0.314, 0.487)	0.392 (0.345, 0.443)	0.158 (0.148, 0.175)	0.827 (0.805, 0.865)

Normalized to attenuation values at t = 0. Values represent medians of data set with minimum and maximum shown in parenthesis.

### Dynamics of optical clearing varied among OCAs

**Fig 4:**
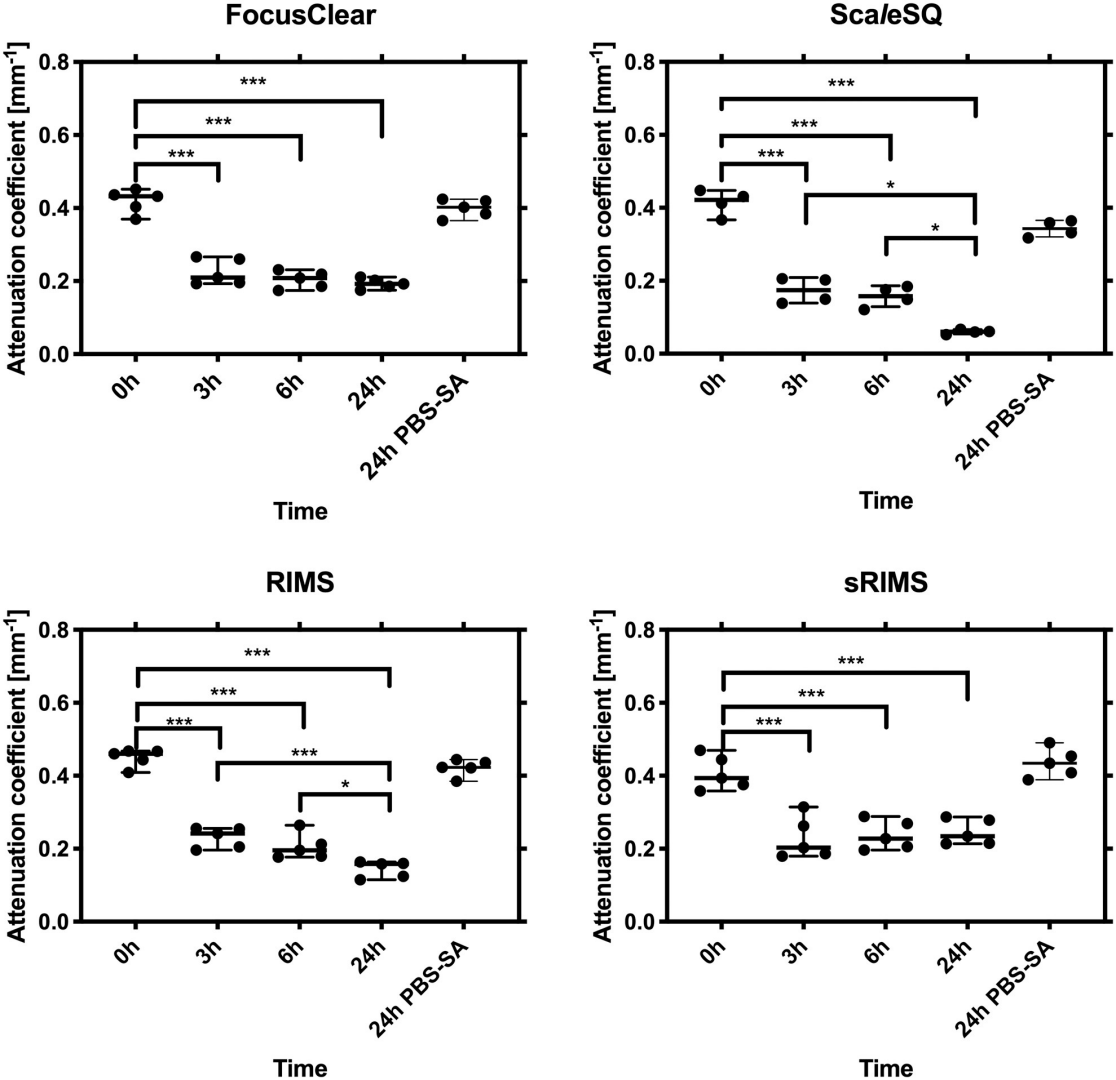
Attenuation reduction significance between time points. Immersion in each optical clearing agent led to a significant reduction in attenuation coefficient. * and *** denote p<0.05 and p<0.001, respectively. PBS-SA = solution of PBS and sodium azide.

We hypothesized that immersion of mouse brain sections in OCAs would decrease light attenuation through the samples. Additionally, prolonged immersion would decrease attenuation further. Fig 4 depicts results from repeated measures ANOVA and Holm-Sidak’s multiple comparison tests were performed. Immersion in each OCA led to a significant decrease in attenuation through the samples when compared to attenuation at t = 0 (p<0.001 in all cases). However, the significance of attenuation reduction among 3, 6, and 24h vary for each agent. A significant decrease in attenuation was observed for Sca*l*eSQ and RIMS comparisons of 3h and 6h to 24h.

### Sca*l*eSQ and RIMS had a significantly greater optical clearing potential than FocusClear and sRIMS

**Fig 5:**
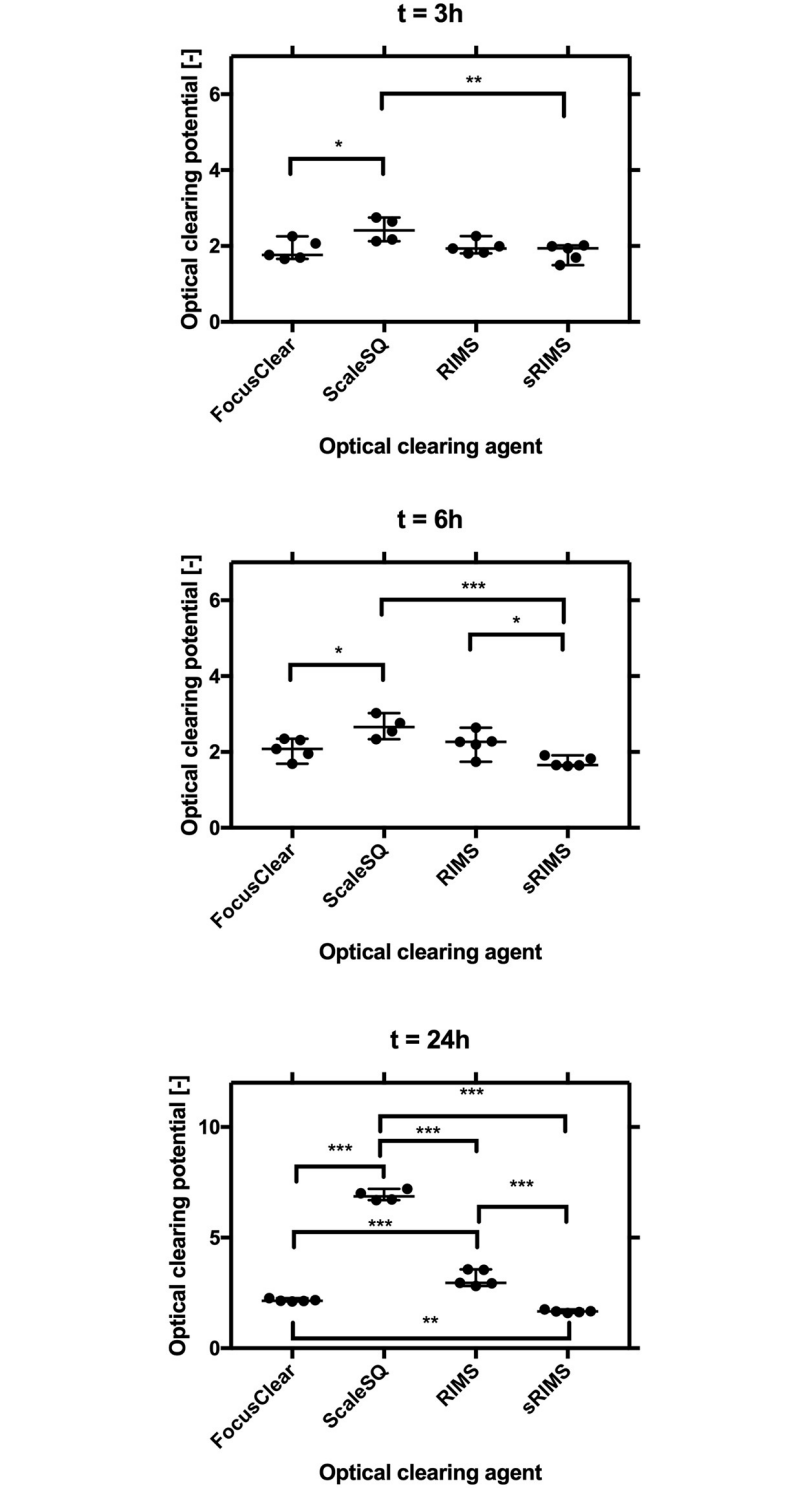
Attenuation reduction significance between OCAs. Of the four optical clearing agents, ScaleSQ had the highest and sRIMS the lowest optical clearing potential at each evaluated time point (3, 6, and 24h). *, **, and *** denote p<0.05, p<0.01, and p<0.001, respectively.

We then hypothesized that the magnitude of attenuation reduction would vary across OCAs. Ordinary one-way ANOVA and Holm-Sidak’s multiple comparison tests were performed for evaluation. As depicted in Fig 5, significant differences during 3h and 6h were only found between Sca*l*eSQ and the other OCAs. However, by 24h differences in optical clearing potential were found throughout all comparisons (p<0.01 for FocusClear/sRIMS and p<0.001 for all else).

### Optical clearing effects of OCAs are reversible

After a total 24 hours of immersion in an OCA, each sample was re-immersed in the initial storage condition of PBS-sodium azide. After 24 hours of re-immersion in PBS-sodium azide, the sample attenuation coefficient increased (p<0.001 for each OCA). The 0h attenuation coefficients of samples that then were immersed in FocusClear, were equivalent to the values after re-immersion in PBS-sodium azide (p = 0.25). For the other OCAs, the 0h attenuation coefficients and the values after re-immersion were slightly different (p≤0.02).

### Sca*l*eSQ enabled deeper microscopic imaging of the cerebral microvasculature, albeit with an apparent decrease in microvascular density

**Fig 6:**
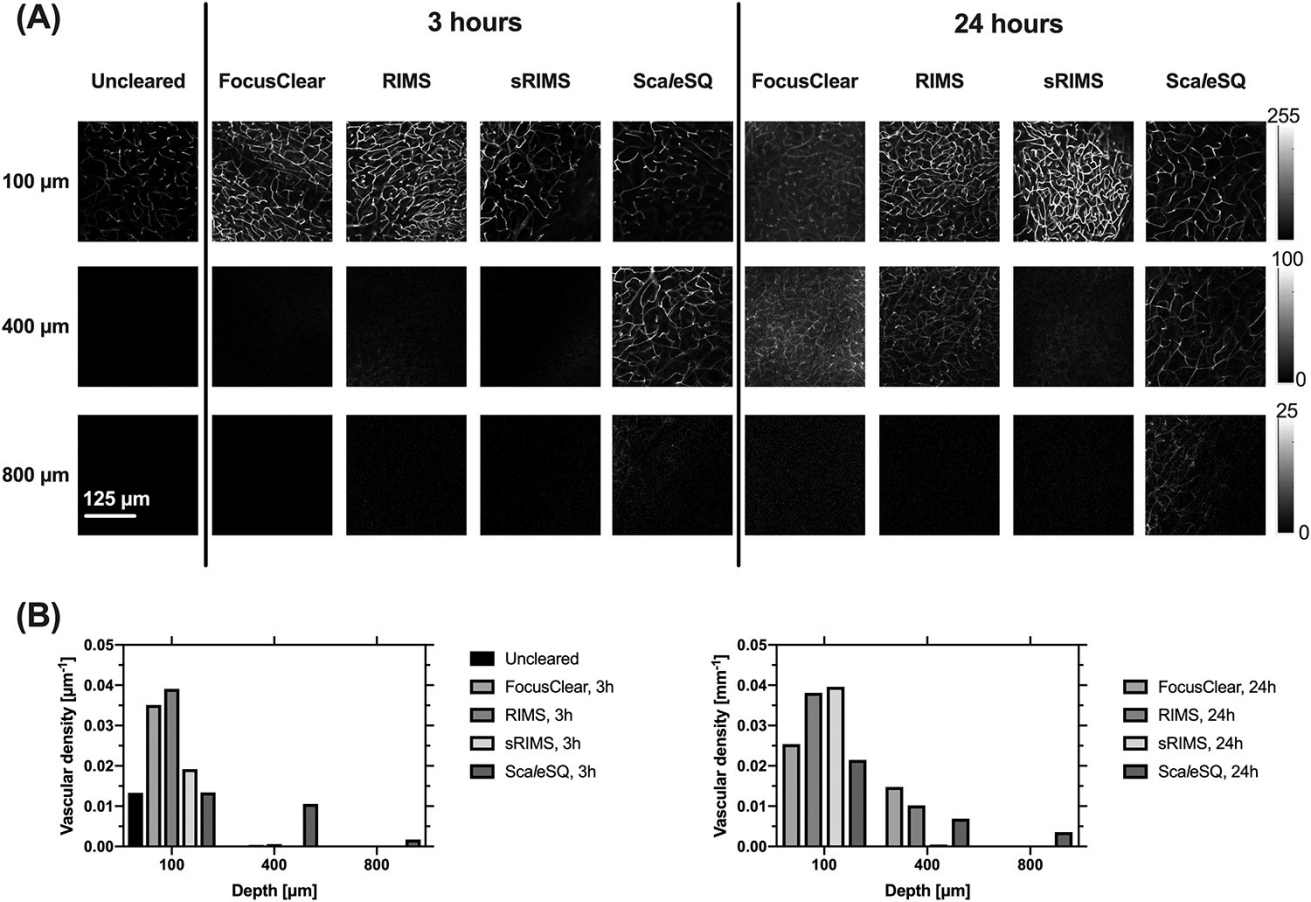
(A) Representative confocal fluorescence microscope scans and (B) vascular density at specific immersion time points and at three imaging depths. Note that the colorbar differs for each row of images, to facilitate comparison of the visibility of microvasculature among clearing agents. Only ScaleSQ enables visualization of microvessels at 800μm depth.

Imaging depth after 24 hours of clearing was consistently greater than after 3 hours of clearing (Fig 6). Sca*l*eSQ showed the greatest achievable imaging depth at both 3h and 24h, with some vasculature evident at 800μm depth. With Sca*l*eSQ immersion, the microvascular density was lower at 100μm, as compared with samples treated with other OCAs.

## Discussion

Our results reveal that immersion of brain sections in specific OCAs affects both their transparency and volume; the optical clearing effect occurs over the entire visible spectrum and is reversible; and that Sca*l*eSQ had the highest optical clearing potential and increase in imaging depth of the four evaluated OCAs, albeit with the largest change in sample volume and a concomitant decrease in apparent microvascular density of the sample.

An increase in sample transparency is an obvious feature of optical clearing. Quantitation of optical clearing effects is necessary to arrive at a true comparison of different approaches [21,23,24]. To maximize comparison of data from the four OCAs evaluated in this study, we divided coronal brain sections into four smaller sections and randomly selected a different OCA to apply to each of these smaller sections. Due to the small sample sizes, we adopted a simpler approach to estimate an optical attenuation coefficient than the dual integrating sphere setup we previously used to study FocusClear [19]. Our current data show a decrease in optical attenuation coefficient with wavelength, which is similar to the scattering trend we and others have shown previously [19,20]. It is important to note that our attenuation estimates are not a true optical attenuation coefficient, which is equal to the sum of the absorption and scattering coefficients of the sample.

We focused our study on comparison of FocusClear with three OCAs (RIMS, sRIMS, and Sca*l*eSQ) that also can be used with simple immersion of the sample. Our data at 640nm (selected as a representative wavelength) demonstrate that the OCAs have different temporal clearing effects. The OCP of FocusClear and sRIMS reached a plateau at 3h, while the OCP of Sca*l*eSQ and RIMS continued to decrease over the 24h clearing period. Our FocusClear OCP observations differed from our previous study [19], in which we observed a significant increase in OCP from 3h to 6h. A potential explanation is the difference in ambient temperature during immersion in the OCA. In our previous work, the temperature was ~25°C, while we performed experiments at 37°C in the current study. The higher temperature may have increased the rate of mass diffusion of FocusClear into the sample and thus led to a faster saturation of the optical clearing effect.

At 24h, the OCP associated with Sca*l*eSQ and RIMS were significantly greater than those of FocusClear and sRIMS, and the OCP of Sca*l*eSQ was nearly twice as high as the OCP of RIMS. Hence, our results suggest that Sca*l*eSQ is the best OCA of the four, in terms of tissue transparency. Our microscopy data are in agreement with the OCP measurements, as the imaging depth achieved with optical clearing trends with increased OCP. It is important to note that, with these OCAs, changes in sample volume also were observed. FocusClear, RIMS, and sRIMS each induced a reduction in volume, while Sca*l*eSQ induced an increase in volume. Reports of volume changes associated with optical clearing in general exist in the literature [16]. Out of the four immersion-based OCAs used in this study, quantitative data exists only for Sca*l*eSQ; Wan et al [24] also observed an increase in sample volume. These volume changes should be considered during interpretation of imaging data. For example, with Sca*l*eSQ, we observed both an increase in imaging depth with immersion time and a decrease in microvascular density. The increase in volume most likely decreases the density of structures that can scatter light, as well as the microvasculature. Such changes may alter quantitative interpretation of the data (i.e., quantification of cerebral microbleed distance from nearby blood vessels [5].

In previous work on optical clearing of unfixed skin samples, we demonstrated that optical clearing potential high for sorbitol due primarily to its high collagen solubility [25], and that chemical fixation of skin negates the ability of hyperosmotic OCAs to achieve clearing [26]. Design of OCA formulations for clearing of fixed tissues involves two primary constituents: 1) an index-matching agent and 2) a detergent that removes lipids. The increased effectiveness of Sca*l*eSQ over the other OCAs tested is likely due to the combination of sorbitol, urea, and Triton X-100. Compared to sRIMS, which uses a high concentration of only sorbitol (index-matching agent), Sca*l*eSQ combines sorbitol with a small quantity of Triton X-100 to remove lipids. The composition of Sca*l*eSQ also suggests the reasons for volume expansion. Although sorbitol is a hyperosmotic agent that would remove tissue shrinkage, the high concentration of urea induces hydration and hence tissue expansion that apparently counteracts the sorbitol dehydrating effects.

Over the 420-720nm wavelength range interrogated in this study, the optical clearing effect in general is more pronounced at longer wavelengths. The shortest typical excitation wavelength used for vessel painting is 488nm, for excitation of fluorescein isothiocyanate (FITC). In our previous work [19], we estimated baseline mouse brain reduced scattering coefficients of 2.62mm^-1^ at 485nm and 1.93mm^-1^ at 642nm. In the present study, at 24h of optical clearing, the median difference in normalized attenuation coefficient between 490 and 640nm wavelengths was ~25%, suggesting that the vascular imaging depth at ~640nm would be nearly twice that at ~488nm. However, it is important to note that imaging depth is not the only consideration for selection of a vessel painting agent, as the quality of vessel labeling differs for different fluorescent dyes [27,28]. Future work should focus on study of both vessel painting approaches and optical clearing approach.

Another relevant factor, specifically for experiments requiring large quantities of OCA, is the cost per volume of OCA used in a given experiment. With the thick sections used in this study, clearing a single sample ranged from approximately $0.06 (Sca*l*eSQ) to upwards of $20 (FocusClear). RIMS and sRIMS also boast lower costs (~$3 and $0.20 respectively) with comparable OCP to FocusClear. Researchers may wish to consider the ratio of price per sample cleared when designing procedures and selecting an OCA that is best suited for their experimental design.

Our study has limitations. Here we assess only four OCAs paired with a single fluorescent labeling method. As many options exist for both optical clearing approaches and vascular fluorescence labeling, care must be taken in extrapolating our findings to other combinations of OCAs and labeling. Here, we focused on brain sections instead of intact brains, so differences in trends may exist if the latter were used as samples [24]. Optimal clearing time often varies among OCAs, ranging from one hour to multiple days, so a more complete timeline of the clearing processes may reveal additional details regarding peak effectiveness.

## Conclusions

In summary, our results indicate that immersion of mouse brain sections in FocusClear, RIMS, sRIMS, or Sca*l*eSQ lead to a decrease in optical attenuation coefficient over the visible spectrum, demonstrate the spatiotemporal dynamics associated with sample immersion, and show that confocal microscopy imaging depth trends with optical clearing potential. We present a rational, quantitative framework for screening and characterization of the impact of optical clearing. Selection of an OCA tailored to the requirements of a given set of experiments should help streamline experimental design and enable a cost-benefit assessment.

## Supporting information

S1 Fig Sample expansion and contraction occur in an anisotropic manner. For each sample and each timepoint, the lateral change in area is plotted versus the measured change in sample thickness. If expansion and contraction occurred in an isotropic manner, the data should fall on a parabola. However, a best-fit quadratic model is associated with a R^2^ value of 0.24, suggesting that volume changes do not occur in an isotropic manner.
(TIFF)

## References

[1] Hirshburg J, Choi B, Nelson J, Yeh A. Correlation between collagen solubility and skin optical clearing using sugars. Lasers in Surgery and Medicine. 2007;39(2):140–144. 10.1002/lsm.20417 17311267

[2] Chung K, Wallace J, Kim S, Kalyanasundaram S, Andalman A, Davidson T et al Structural and molecular interrogation of intact biological systems. Nature. 2013;497(7449):332–337. 10.1038/nature12107 23575631 PMC4092167

[3] Lagerweij T, Dusoswa S, Negrean A, Hendrikx E, de Vries H, Kole J et al Optical clearing and fluorescence deep-tissue imaging for 3D quantitative analysis of the brain tumor microenvironment. Angiogenesis. 2017;20(4):533–546. 10.1007/s10456-017-9565-6 28699046 PMC5660146

[4] Hama H, Hioki H, Namiki K, Hoshida T, Kurokawa H, Ishidate F et al ScaleS: an optical clearing palette for biological imaging. Nature Neuroscience. 2015;18(10):1518–1529. 10.1038/nn.4107 26368944

[5] Lo P, Crouzet C, Vasilevko V, Choi B. Visualization of microbleeds with optical histology in mouse model of cerebral amyloid angiopathy. Microvascular Research. 2016;105:109–113. 10.1016/j.mvr.2016.02.002 26876114 PMC4814270

[6] Moy A, Wiersma M, Choi B. Optical Histology: A Method to Visualize Microvasculature in Thick Tissue Sections of Mouse Brain. PLoS ONE. 2013;8(1):e53753 10.1371/journal.pone.0053753 23372668 PMC3553090

[7] Marx V. Microscopy: seeing through tissue. Nature Methods. 2014;11(12):1209–1214. 10.1038/nmeth.3181 25423017

[8] Belle M, Godefroy D, Dominici C, Heitz-Marchaland C, Zelina P, Hellal F et al A Simple Method for 3D Analysis of Immunolabeled Axonal Tracts in a Transparent Nervous System. Cell Reports. 2014;9(4):1191–1201. 10.1016/j.celrep.2014.10.037 25456121

[9] Ke M, Fujimoto S, Imai T. SeeDB: a simple and morphology-preserving optical clearing agent for neuronal circuit reconstruction. Nature Neuroscience. 2013;16(8):1154–1161. 10.1038/nn.3447 23792946

[10] Ku T, Swaney J, Park J, Albanese A, Murray E, Cho J et al Multiplexed and scalable super-resolution imaging of three-dimensional protein localization in size-adjustable tissues. Nature Biotechnology. 2016;34(9):973–981. 10.1038/nbt.3641 27454740 PMC5070610

[11] Susaki E, Tainaka K, Perrin D, Kishino F, Tawara T, Watanabe TM, et al Whole-brain imaging with single-cell resolution using chemical cocktails and computational analysis. Cell. 2014;157:726–739. 10.1016/j.cell.2014.03.042 24746791

[12] Costantini I, Ghobril J, Di Giovanna A, Allegra Mascaro A, Silvestri L, Mullenbroich M, et al A versatile clearing agent for multi-modal brain imaging. Scientific Reports. 2015;5:9808. 25950610 10.1038/srep09808 PMC4423470

[13] Yu T, Zhu J, Li Y, Ma Y, Wang J, Cheng X, et al RTF: a rapid and versatile tissue optical clearing method. Scientific Reports, 2018;8(1):1964 10.1038/s41598-018-20306-3 29386656 PMC5792593

[14] Hou B, Zhang D, Zhao S, Wei M, Yang Z, Wang S, et al Scalable and DiI-compatible optical clearance of the mammalian brain. Frontiers in Neuroanatomy. 2015;9:1–19. 25759641 10.3389/fnana.2015.00019 PMC4338786

[15] Yu T, Qi Y, Gong H, Luo Q, Zhu D. Optical clearing for multiscale biological tissues. Journal of Biophotonics, 2018,11(2):e201700187. 10.1002/jbio.201700187 29024450

[16] Li Y, Song Y, Zhao L, Gaidosh G, Laties A, Wen R. Direct labeling and visualization of blood vessels with lipophilic carbocyanine dye DiI. Nature Protocols. 2008;3(11):1703–1708. 10.1038/nprot.2008.172 18846097 PMC2811090

[17] Yardeni T, Eckhaus M, Morris H, Huizing M, Hoogstraten-Miller S. Retro-orbital injections in mice. Lab Animal. 2011;40(5):155–160. 10.1038/laban0511-155 21508954 PMC3158461

[18] Moy A, Lo P, Choi B. High-resolution visualization of mouse cardiac microvasculature using optical histology. Biomedical Optics Express. 2013;5(1):69 10.1364/BOE.5.000069 24466477 PMC3891346

[19] Moy A, Capulong B, Saager R, Wiersma M, Lo P, Durkin A et al Optical properties of mouse brain tissue after optical clearing with FocusClear^™^. Journal of Biomedical Optics. 2015;20(9):095010. 10.1117/1.JBO.20.9.095010 PMC4963466 26388460

[20] d’Esposito A, Nikitichev D, Desjardins A, Walker-Samuel S, Lythgoe M. Quantification of light attenuation in optically cleared mouse brains. Journal of Biomedical Optics. 2015;20(8):080503. 10.1117/1.JBO.20.8.080503 PMC4568291 26277988

[21] Choi B, Tsu L, Chen E, Ishak T, Iskandar S, Chess S et al Determination of chemical agent optical clearing potential using in vitro human skin. Lasers in Surgery and Medicine. 2005;36(2):72–75. 10.1002/lsm.20116 15666319

[22] White S, George S, Choi B. Automated computation of functional vascular density using laser speckle imaging in a rodent window chamber model. Microvascular Research. 2011;8:92–95. 10.1016/j.mvr.2011.03.006 PMC3137926 21419785

[23] Magliaro C, Callara A, Mattei G, Morcinelli M, Viaggi C, Vaglini F et al Clarifying CLARITY: Quantitative Optimization of the Diffusion Based Delipidation Protocol for Genetically Labeled Tissue. Frontiers in Neuroscience. 2016;10. 27199642 10.3389/fnins.2016.00179 PMC4847491

[24] Wan P, Zhu J, Xu J, Li Y, Yu T, Zhu D. Evaluation of seven optical clearing methods in mouse brain. Neurophotonics. 2018;5(03):1. 10.1117/1.NPh.5.3.035007 PMC6109056 30155510

[25] Hirshburg J, Choi B, Nelson J, Yeh A. Collagen solubility correlates with skin optical clearing. Journal of Biomedical Optics. 2006;11(4):040501 10.1117/1.2220527 16965124

[26] Yeh A, Choi B, Nelson J, Tromberg B. Reversible dissociation of collagen in tissues. Journal of Investigative Dermatology. 2003;121(6):1332–1335. 10.1046/j.1523-1747.2003.12634.x 14675178

[27] Konno A, Matsumoto N, Okazaki S. Improved vessel painting with carbocyanine dye-liposome solution for visualisation of vasculature. Scientific Reports. 2017;7(1):10089 10.1038/s41598-017-09496-4 28855543 PMC5577039

[28] Di Giovanna A, Tibo A, Silvestri L, Mullenbroich M, Costantini I, Allegra Mascaro A et al Whole-Brain Vasculature Reconstruction at the Single Capillary Level. Scientific Reports. 2018;8(1):12573 10.1038/s41598-018-30533-3 30135559 PMC6105658

