# Supplementary figures and images for: Optical clearing potential of immersion-based agents applied to thick mouse brain sections
Source: PLoS One. 2019 May 10;14(5):e0216064. doi: 10.1371/journal.pone.0216064 (PMC6510422; doi:10.1371/journal.pone.0216064)

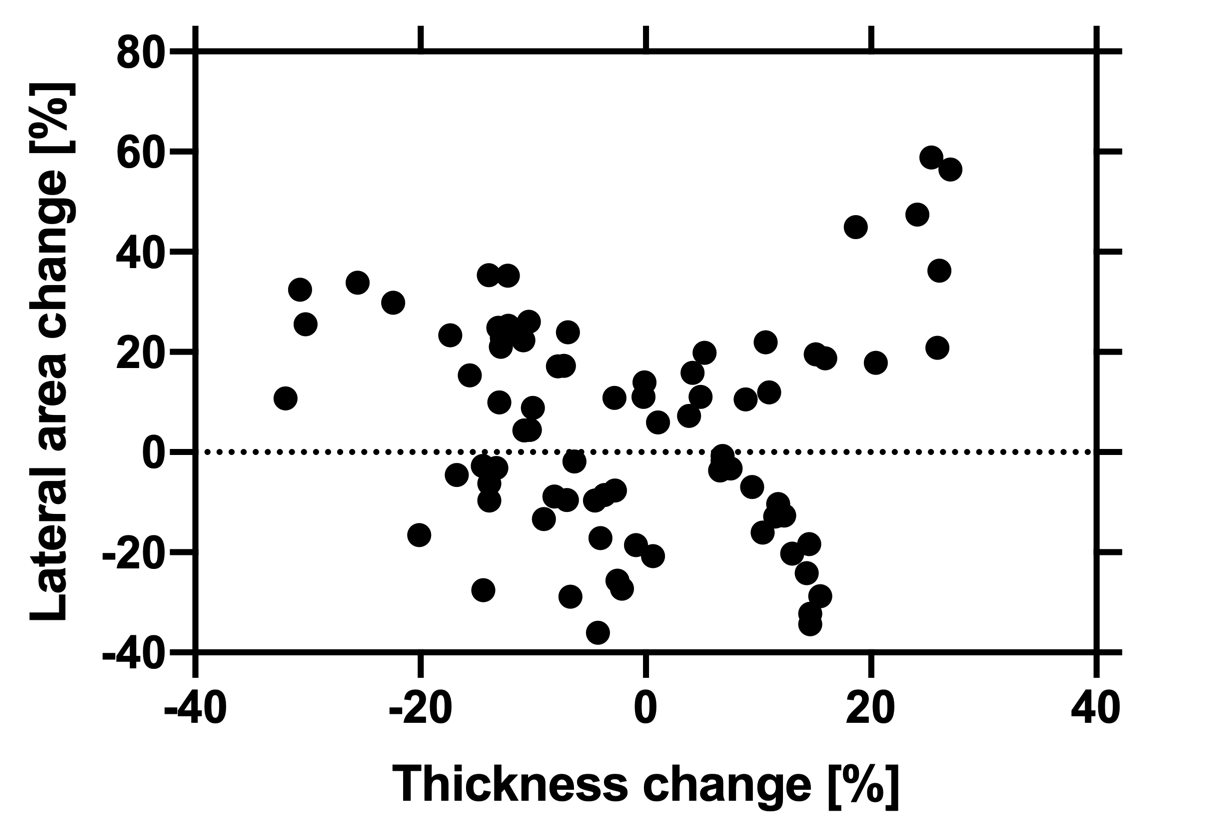

Supplement: S1 Fig — For each sample and each timepoint, the lateral change in area is plotted versus the measured change in sample thickness. If expansion and contraction occurred in an isotropic manner, the data should fall on a parabola. However, a best-fit quadratic model is associated with a R2 value of 0.24, suggesting that volume changes do not occur in an isotropic manner. (TIFF) [file pone.0216064.s001.tiff]
